# Air Quality Modeling in Support of the Near-Road Exposures and Effects of Urban Air Pollutants Study (NEXUS)

**DOI:** 10.3390/ijerph110908777

**Published:** 2014-08-27

**Authors:** Vlad Isakov, Saravanan Arunachalam, Stuart Batterman, Sarah Bereznicki, Janet Burke, Kathie Dionisio, Val Garcia, David Heist, Steve Perry, Michelle Snyder, Alan Vette

**Affiliations:** 1 National Exposure Research Laboratory, United States Environmental Protection Agency, 109 T.W. Alexander Drive, Research Triangle Park, NC 27711 USA; E-Mails: sarah.bereznicki@gmail.com (S.B.); Burke.janet@epa.gov (J.B.); Dionisio.Kathie@epa.gov (K.D.); Garcia.Val@epa.gov (V.G.); Heist.David@epa.gov (D.H.); Perry.Steven@epa.gov (S.P.); Vette.Alan@epa.gov (A.V.); 2 Institute for the Environment, University of North Carolina at Chapel Hill, 100 Europa Drive, Chapel Hill, NC 27517, USA; E-Mails: sarav@unc.edu (S.A.); mgs3584@email.unc.edu (M.S.); 3 Department of Environmental Health Sciences, School of Public Health, University of Michigan, Room 6075 SPH2, 1420 Washington Heights, Ann Arbor, MI 48109-2029 USA; E-Mail: stuartb@umich.edu

**Keywords:** dispersion modeling, air pollution, exposure, traffic

## Abstract

A major challenge in traffic-related air pollution exposure studies is the lack of information regarding pollutant exposure characterization. Air quality modeling can provide spatially and temporally varying exposure estimates for examining relationships between traffic-related air pollutants and adverse health outcomes. A hybrid air quality modeling approach was used to estimate exposure to traffic-related air pollutants in support of the Near-Road Exposures and Effects of Urban Air Pollutants Study (NEXUS) conducted in Detroit (Michigan, USA). Model-based exposure metrics, associated with local variations of emissions and meteorology, were estimated using a combination of the American Meteorological Society/Environmental Protection Agency Regulatory Model (AERMOD) and Research LINE-source dispersion model for near-surface releases (RLINE) dispersion models, local emission source information from the National Emissions Inventory, detailed road network locations and traffic activity, and meteorological data from the Detroit City Airport. The regional background contribution was estimated using a combination of the Community Multi-scale Air Quality (CMAQ) and the Space-Time Ordinary Kriging (STOK) models. To capture the near-road pollutant gradients, refined “mini-grids” of model receptors were placed around participant homes. Exposure metrics for CO, NO*_x_*, PM_2.5_ and its components (elemental and organic carbon) were predicted at each home location for multiple time periods including daily and rush hours. The exposure metrics were evaluated for their ability to characterize the spatial and temporal variations of multiple ambient air pollutants compared to measurements across the study area.

## 1. Introduction

Studies of health effects associated with exposure to traffic-related air pollutants have typically used surrogates of exposure, such as residential proximity to roadways, traffic volumes on nearby roadways, and land-use regression techniques, to estimate exposure for the study population [1,2,3,4,5,6]. While these exposure metrics are relatively simple to generate and have minimal data requirements, they do not capture potentially important influences on spatial variability, and perhaps more importantly, temporal variability of traffic-related air pollutants such as factors that affect dispersion [7]. Traffic-related air pollutants can have significant temporal variability due to traffic activity patterns (e.g., rush hour peaks, higher during weekdays *vs.* weekends), emission profiles that vary with temperature, and the influence of meteorology, which are not captured by static exposure estimates based on geographic parameters (*i.e.*, proximity to roadway, traffic intensity, lane use, *etc.*) that are often used in traffic studies.

Health studies of the effects of traffic-related pollutants have historically relied on exposure metrics such as those listed above because available central site measurements often do not adequately capture local influences from traffic. Data from regulatory monitoring sites may capture temporal variations for some pollutants (e.g., NO*_x_*, CO), but spatial coverage within an urban area is generally limited to one or two sites. Studies deploying multiple monitors to provide spatial coverage are costly, so samplers with lower temporal resolution (daily to weekly) are often used [8,9]. The spatial impact of traffic emissions also varies by pollutant due to their chemical and physical characteristics [10], therefore a number of different monitors are needed to obtain data for the various traffic-related air pollutants.

To address the limitations of available monitoring data and the various metrics of exposure, recent studies have utilized emission/dispersion models and daily activity locations to derive air pollution exposures for epidemiological studies [11,12,13,14,15,16,17]. Two main types of air quality models are relevant for this purpose: grid-based chemical transport models and plume dispersion models. Grid-based chemical transport models, such as the Community Multiscale Air Quality (CMAQ) model, estimate concentrations for large geographic areas at high time resolution but cannot resolve features smaller than a grid cell, usually several kilometers across [18]. Plume dispersion models, such as American Meteorological Society/Environmental Protection Agency Regulatory Model (AERMOD) [19], can provide locally resolved concentration gradients such as those occurring close to roadways but require estimates of background concentrations to compare model results to measurement data [20]. To account for the limitations of each type of model, a hybrid approach can be used where output from both a grid-based chemical transport model and a plume dispersion model are merged to provide contributions from photochemical interactions, long-range (regional) transport, and details attributable to local-scale dispersion [21,22].

The Near-road Exposures and Effects of Urban Air Pollutants Study (NEXUS) is investigating the respiratory health impacts of exposure to traffic-related air pollutants for children with asthma living near major roads in Detroit, MI [23]. Air quality modeling was included in the design of NEXUS to estimate exposure to traffic-related air pollutants that varied both spatially and temporally. Exposure estimates will be used for evaluating associations with daily health measurements collected during a 14-day period in each of four seasons for each study participant over a 1.5 years period. This paper describes application of the hybrid air quality modeling approach. The hybrid modeling components are described along with the specific inputs used for application to the Detroit study area and NEXUS participant locations. Model results are compared with available measurement data from regulatory monitoring sites within Detroit and intensive field studies conducted during NEXUS. The various exposure metrics produced from the model output which include the mobile source contribution to total exposure are provided for use in related NEXUS epidemiologic analysis, and described and compared here.

## 2. Air Quality Modeling Approach for Estimating Exposure Metrics

We use a combination of local-scale dispersion models, regional-scale models and observations to provide temporally and spatially-resolved pollutant concentrations for the epidemiologic analysis. Local variations in emissions and meteorology were estimated using a combination of AERMOD and RLINE [24,25] dispersion models. RLINE is a research-level, line-source dispersion model developed by U.S. EPA’s Office of Research and Development as a part of the ongoing effort to further develop tools for a comprehensive evaluation of air quality impacts in the near-road environment. This model incorporates traffic activity and primary mobile source emissions estimates to model hourly exposures to traffic emissions for the NEXUS participants. Exposures to air pollution from stationary sources such as manufacturing facilities and other non-road mobile sources were modeled using AERMOD. The input data including the source locations, emission rates, source parameters and other information were obtained from the 2008 official version of the National Emissions Inventory (NEI) from the U.S. EPA, the latest available at the time of the study [26].

To generate the total exposure of the NEXUS study participants, the urban background contribution must be added to the local estimates of exposure provided by AERMOD and RLINE models. The background contribution was estimated using a combination of the Community Multiscale Air Quality (CMAQ) model and the Space/Time Ordinary Kriging (STOK) model [27]. Two CMAQ model simulations were conducted: the baseline simulation represented all emissions in a broad region (covering the eastern US); the second removed all anthropogenic emissions in the NEXUS study domain. The ratios of concentrations predicted by CMAQ in these two simulations in the Detroit region along with measurements from the routine observational network in the region were used to estimate background pollutant concentrations at the NEXUS study locations.

**Figure 1:**
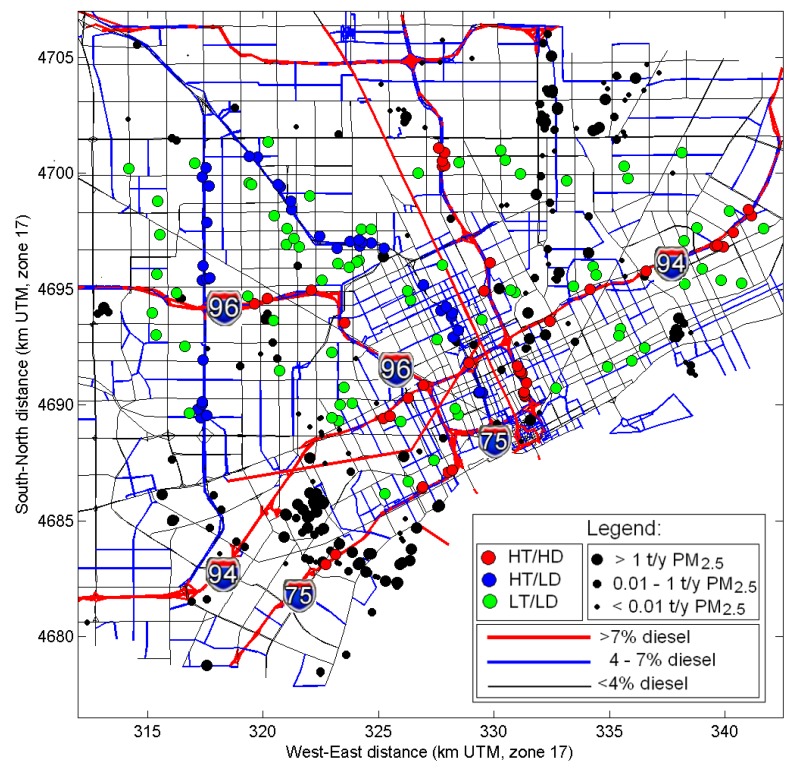
Modeling domain for the NEXUS study. Major highways are shown as red and blue lines (for >7% diesel and 4%–7% diesel fraction) and other roads–as black lines. Model receptors are shown in red, blue and green circles for the HD, LD and LT traffic exposure group, respectively. Stationary sources are shown as black dots (symbol size indicates the magnitude of PM_2.5_ annual emissions).

The modeling provided hourly pollutant concentrations for CO, NO*_x_*, total PM_2.5_ mass, and its components such as elemental carbon (EC) and organic carbon (OC), and benzene. Hourly concentrations were processed to calculate daily and annual average exposure metrics for each study participants’ home and school location. The model-based exposure metrics provided the necessary inputs for use in the epidemiologic analyses to determine if children in Detroit, MI with asthma living in close proximity to major roadways have greater health impacts associated with traffic-related air pollutants than those living farther away, particularly for children living near roadways with high diesel traffic. Children were recruited on the basis of the proximity of their residence to roadways in three exposure groups: children living within 150 m of high traffic and high diesel (HD) roads, defined as having traffic that exceeds 6000 commercial vehicles/day (commercial annual average daily traffic; CAADT) and 90,000 total vehicles/day (annual average daily traffic; AADT); children living within 150 m of high traffic low diesel (LD) road, defined similarly but only including roads with CAADT below 4500; and children living in low traffic (LT) areas, defined as at least 300 m from any road with over 25,000 AADT (Figure 1).

We first estimated pollutant-specific local-scale air concentrations for stationary and area sources using AERMOD. This model utilized information on local emission sources for these two sectors and local meteorological conditions to estimate hourly average concentrations at multiple receptors in each of the three exposure groups. Emission data for major stationary sources and airport sources were obtained from the NEI. For mobile sources, we used a recently developed line source dispersion model RLINE [24,25]. Roadway emissions were estimated using detailed road network locations and a bottom-up methodology for roadway emissions [20], and further elaborated in [28].

An analysis of wind patterns for the year 2010 based on hourly meteorological observations from the NWS stations within and around the study area (Detroit City airport, Detroit Metro airport, Windsor airport) determined that the Detroit-City airport station was most representative of the NEXUS modeling domain, and which also had the most data completeness objective. Hourly surface observations from Detroit City, in combination with data from the nearest upper air station (DTX-72632 Oakland County) were used for the simulation period to drive the modeling. The land characteristics around the station were determined and the AERSURFACE model was applied. The AERMET program was used to process the meteorological data from the Detroit City airport and DTX upper air station for input into AERMOD.

Emissions within the 30 × 40 km source region centered on the NEXUS participants in Detroit were extracted from the NEI 2008 by major source categories (area, point, onroad and off-road mobile) for the pollutants of interest. Sources located in Macomb, Oakland, and Wayne counties in Michigan, and Essex County in Ontario, Canada were included. Area sources such as port- and airport-type sources in the study area were also included.

For stationary point sources, the location, emission rate, and individual stack parameters (e.g., stack height, exit velocity) were used. Other non-stack emissions (such as smaller sources with no stack parameters, fugitive emissions, and emissions from nonroad mobile sources) were modeled as area sources. County-level NEI area source emissions were spatially re-allocated to 1 km × 1 km grid-cell resolution using spatial surrogates within the SMOKE emissions processor [29]. Airport area sources with a polygon-shaped area corresponding to their actual locations were used as an input to the model. Stationary sources were temporally allocated using SMOKE. The SMOKE processor contains monthly, weekly, diurnal-weekday and diurnal-weekend profiles. A seasonal profile was calculated from the monthly profiles. The final temporal allocation yields an emission rate for each hour of the weekday/Saturday/Sunday for the entire year.

**Figure 2:**
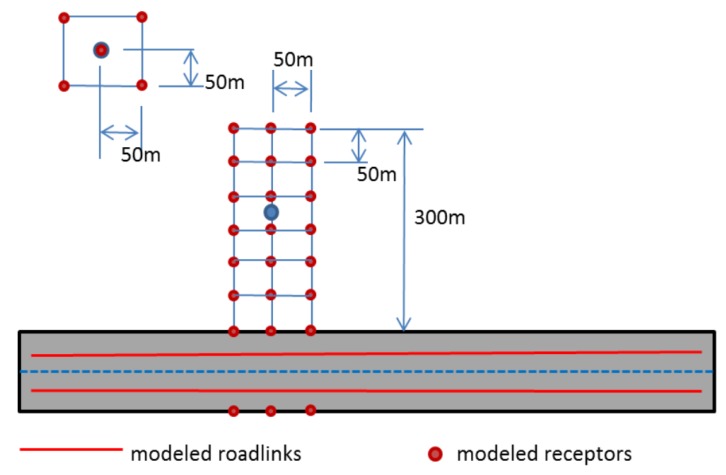
Model receptors near roadways: 24-receptor mini-grid network.

For onroad mobile source emissions, the methodology described in [20] is followed that produces a spatially and temporally resolved mobile source emissions inventory (*i.e*., hourly emissions for all pollutants modeled, by vehicle class and road link). This methodology was successfully applied in previous studies for New Haven, Atlanta and Baltimore [16,22,30]. In this study, detailed information including the geometry of the road network, traffic volumes, temporal allocation factors, fleet mixes and pollutant-specific emission factors, assembled from a variety of sources, were used in combination with meteorological inputs to generate link-based emissions for use in dispersion modeling to estimate pollutant concentrations due to traffic [28]. The total emissions were calculated from emission factors multiplied by traffic activity for each road link to provide inputs for RLINE model simulations across the NEXUS study domain for a 1.5 years period (Fall 2010–Spring 2012). In order to evaluate the differences in near-road pollutant gradients between the three selected traffic exposure groups (low diesel LD, high diesel HD and low traffic LT), the receptor grids were refined within each NEXUS sub-area (including the participants homes and schools). A mini-grid of receptors was placed near each NEXUS participant’s home and school consisting of a rectangular receptor grid on 50 m centers as indicated in Figure 2. Depending on the number of receptors used, mini-grids gave anonymity to 50 or 100 m, a distance sufficient to protect the participants’ identity.

Exposure metrics were calculated from mini-grids to produce estimates for each NEXUS location. For NEXUS locations in the near road group, there are 85 near-road grids. The near-road grids contain 24 modeled receptors, and a weighted interpolation between modeled grid rows was performed based on the actual distance between the participant’s home and the nearest major roadway to estimate the hourly concentration. Other locations were modeled with 5-point receptor grids (using five receptors on and around the home) and the hourly concentration was estimated by taking an average of the modeled concentrations at the five points.

From hourly concentration, exposure metrics were calculated for the following time periods: 24 h (daily); 1–6 (a.m. off-peak); 7–8 (a.m. peak); 9–14 (mid-day); 15–17 (p.m. peak); and 18–24 (p.m. off-peak). These hours correspond to the reported local-time (e.g., hour 1 represents from 12:01 a.m.–1:00 a.m.). These are calculated with a 70% completeness criterion for the hourly meteorology in each time period. These daily exposure metrics for CO, NO*_x_*, PM_2.5_ and its components (EC and OC), capturing spatial and temporal variability across health study domain (Fall 2010–Spring 2012) were used in the epidemiologic analyses.

## 3. Results and Discussion

**Figure 3:**
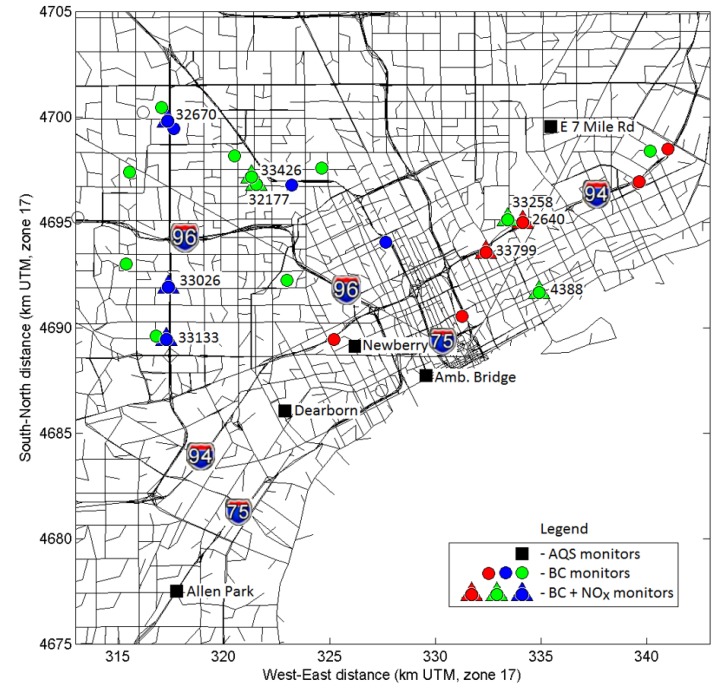
Locations of PM_2.5_, black carbon (BC) and NO*_x_* monitors at NEXUS (●, ▲) and AQS sites (■). (Notes: Colors of symbols denote roadway classification as described in Figure 1; numbers next to the NEXUS site locations indicate measurement site ID).

**Figure 4:**
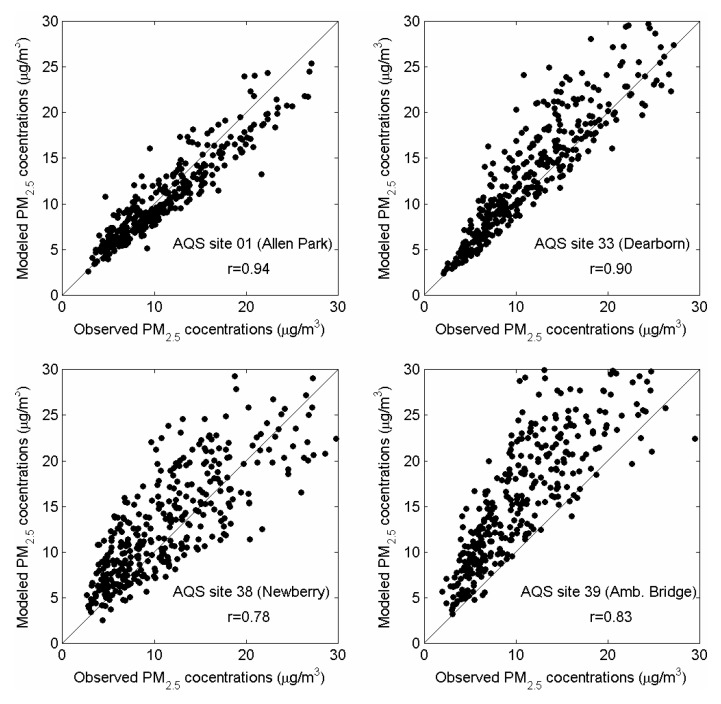
Model to monitor comparison: daily average PM_2.5_ concentrations for one-year period of 2010 at four AQS sites in the Detroit modeling domain.

**Figure 5:**
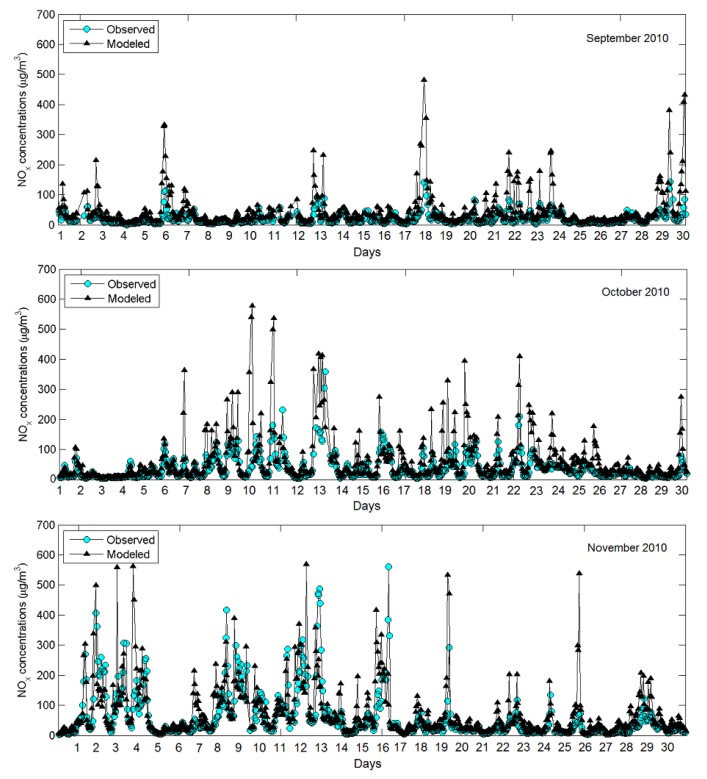
Model to monitor comparison: time series of hourly NO_x_ concentrations at the AQS site 26-163-0019 (E. 7 Mile Road) for three-month period September-November 2010.

Model results were compared to ambient monitoring data in Detroit. There are two sets of monitoring data for model evaluation: (1) from the routine observational network (AQS); and (2) from the intensive monitoring campaign which was part of the NEXUS study. There are five AQS monitoring stations in the modeling domain: four PM_2.5_ monitors (Allen Park, Dearborn, Newberry School, Ambassador Bridge) and one NO_x_ monitor (East 7 mile road), as indicated in Figure 3. A comparison between modeled daily average PM_2.5_ concentrations for one-year period of 2010 at observed PM_2.5_ concentrations at all four AQS sites is shown in Figure 4. Model results correlate well with observed data (r ranges from 0.78 to 0.94) and are generally within a factor of two from observations. The Allen Park site near I-75 and southwest of stationary sources has best comparison *vs.* other sites closer to large sources. There is more scatter at the “Newberry” and “Ambassador Bridge” sites, likely due to uncertainties in spatial allocation of emissions near these locations. These sites are impacted by local emission sources modeled as 1 km × 1 km area sources in AERMOD. In contrast, the “Dearborn” site is impacted by industrial sources modeled as stacks with their known locations. For NO*_x_*, only one monitoring site was available in the modeling domain. The “East 7 mile” site is in the North-Eastern corner of the modeling domain, away from major highways. Figure 5 compares time series of modeled and observed hourly NO*_x_* concentrations at the “East 7 mile” site for September–November 2010. Modeled concentrations generally follow the time series of observed data, however there are some over-predictions at certain hours likely due to uncertainties in emissions from traffic. The monitoring site is away from major highways, therefore the observed concentrations are influenced by emissions from local roads and regional sources. Unlike major highways, estimating emissions from local roads is more challenging because of uncertainties in road locations, traffic activity and fleet distribution. The results of statistical analyses (*i.e*., Mean Bias, Mean Error, R, FAC2) comparing the modeled and measurement data from five AQS monitoring stations in the modeling domain are summarized in Table 1.

**Table 1:** Statistics metrics for the model-to-monitor comparison at the five AQS monitoring stations for PM_2.5_ and NO*_x_*.

Pollutant	PM_2.5_				NO_x_
Site	261630001	261630033	261630038	261630039	261630019
Obs. Mean	10.865	11.694	11.050	11.619	32.656
Model Mean	10.370	13.646	14.233	18.243	62.255
Mean Bias	−0.495	1.952	3.183	6.624	29.598
Mean Error	2.420	4.254	5.798	7.834	35.654
R	0.760	0.624	0.480	0.502	0.515
FAC2	0.965	0.905	0.818	0.787	0.616
Pairs	8365	8438	8297	8455	8100

**Figure 6:**
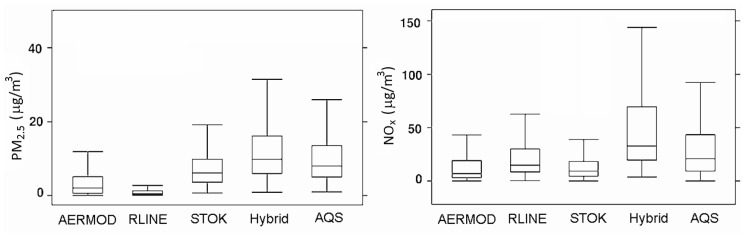
Distributions of modeled and observed PM_2.5_ and NO*_x_* concentrations for 2010 at the AQS monitoring sites. (all four PM_2.5_ averaged, and one NO*_x_* site).

**Figure 7:**
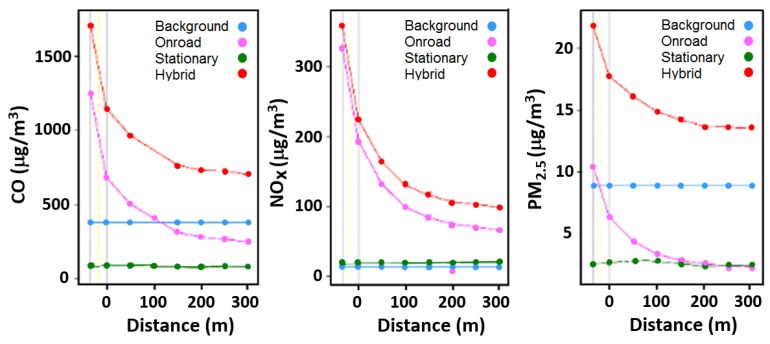
Near-road pollutant gradients of CO, NO*_x_* and PM_2.5_ concentrations (2010 annual average) from a mini-grid of 24 model receptors near the I-94 freeway.

The modeling provides an opportunity to compare the relative contributions of various sources: stationary sources (*i.e*., AERMOD), roadways (*i.e.*, RLINE), urban background (*i.e.*, STOK), and total (Hybrid). Figure 6 compares distributions of modeled and observed concentrations for PM_2.5_ (all four AQS sites combined) and NO*_x_* (one AQS site) for 2010, and also shows relative contributions of various sources. As can be seen from Figure 6, the relative contribution of roadways is very small for PM_2.5_ but quite high for NO*_x_*, whereas urban background is more significant for PM_2.5_ than for NO*_x_*. The difference in relative contribution of roadway emissions to the total concentration between pollutants is further illustrated in Figure 7 using a single receptor site near the I-94 freeway as an example. The model predicts steep gradients of near-road concentrations for all pollutants (CO, NO*_x_* and PM_2.5_) at the modeled receptor site near I-94. However, the background contribution is different for these pollutants. For CO, the roadway contribution is high within 100 m from the roadway, but after 100 m it diminishes to levels below the background. For NO*_x_*, the background is low and roadway impact dominates at this site. For PM_2.5_, the background dominates and primary impact of roadway emissions contributes only about 10%–25% of the total concentration.

**Figure 8:**
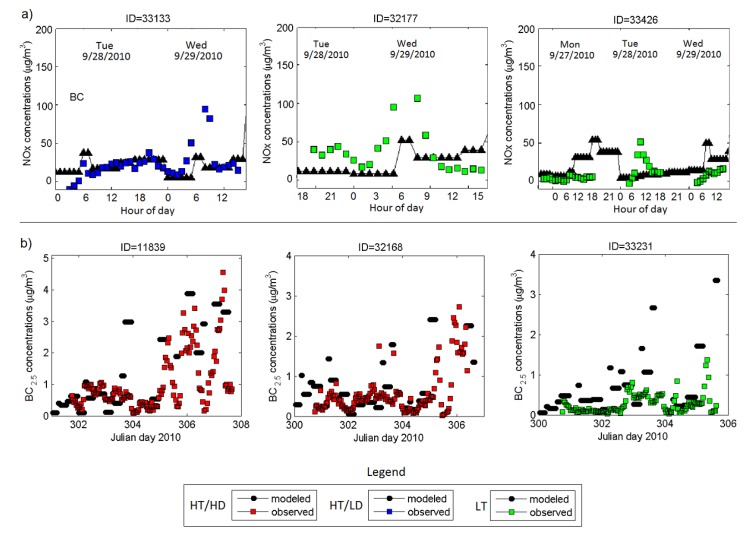
Comparison of modeled exposure metrics and observed concentrations for NO*_x_* at six different NEXUS monitoring sites.

Measurements of air pollutant exposures also have uncertainties, such as from the measurement method or instrument, as well as whether the measurement captures actual air pollutant exposures or is a surrogate for it (e.g., central site monitors). Although the sub-daily modeled exposure metrics may have greater uncertainty than daily or longer-term averages, few monitoring methods exist that can measure exposures with time resolution below daily averages. Collecting limited high-time resolution measurements for comparison with model predictions is one approach to help identify potential contributors to the modeling uncertainty. In addition to observational data from the routine monitoring network, we also used monitoring data from the 2010 intensive monitoring campaign of the NEXUS study. During the September-November 2010 study period, black carbon (BC) measurements were made at 25 NEXUS home locations and NO*_x_* was measured at nine NEXUS homes (Figure 3). Figure 8 compares modeled and observed concentrations at selected NEXUS homes for NO*_x_* and BC. As can be seen from the figure, the model generally captures the time series of observed NO*_x_* concentrations. However, at some sites and for some specific hours, the model under-predicts concentrations (e.g., at site ID = 33,133 or ID = 32,177, 6–8 a.m. on 29 September 2010) or over-predicts (e.g., at site ID = 33,426, 6–8 a.m. on 29 September 2010) concentrations at some locations. This discrepancy can be explained by the uncertainty in hourly traffic activity at the road link level. Typically, time-resolved traffic information at a link level is not available and sophisticated algorithms are used to estimate such traffic emissions for individual road links. Nevertheless, except for some events, the model can capture the magnitude and time patterns of near road pollutant concentrations, critical for the exposure and health studies. For BC, the model performance was similar to NO*_x_*, if not better at the sites shown.

**Figure 9:**
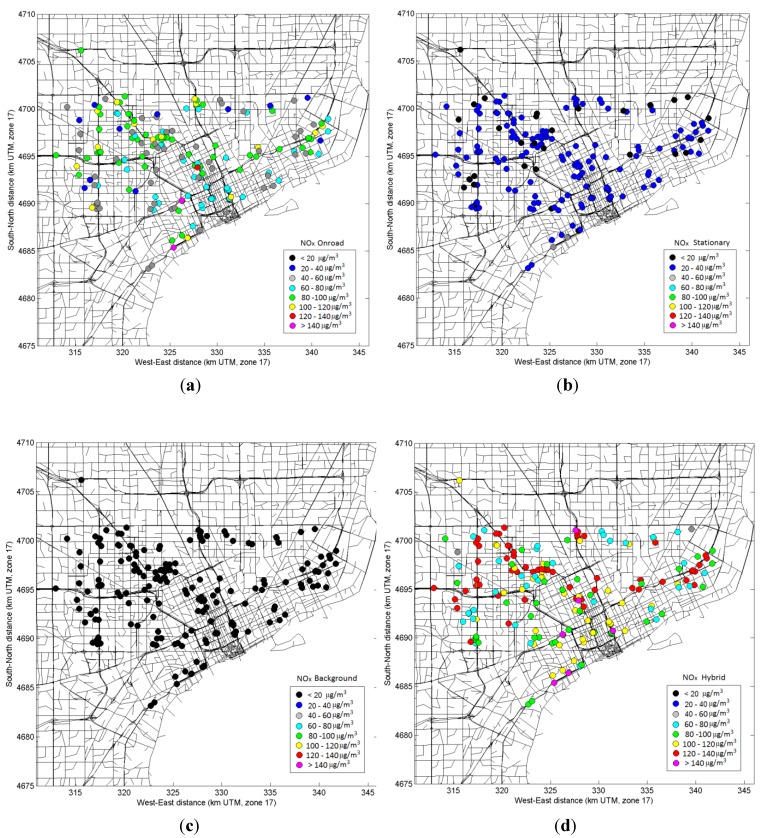
Spatial maps of modeled daily NO_x_ concentrations averaged during September-October 2010, showing contributions from mobile sources (**a**); stationary sources (**b**); urban background (**c**); and total (**d**).

**Figure 10:**
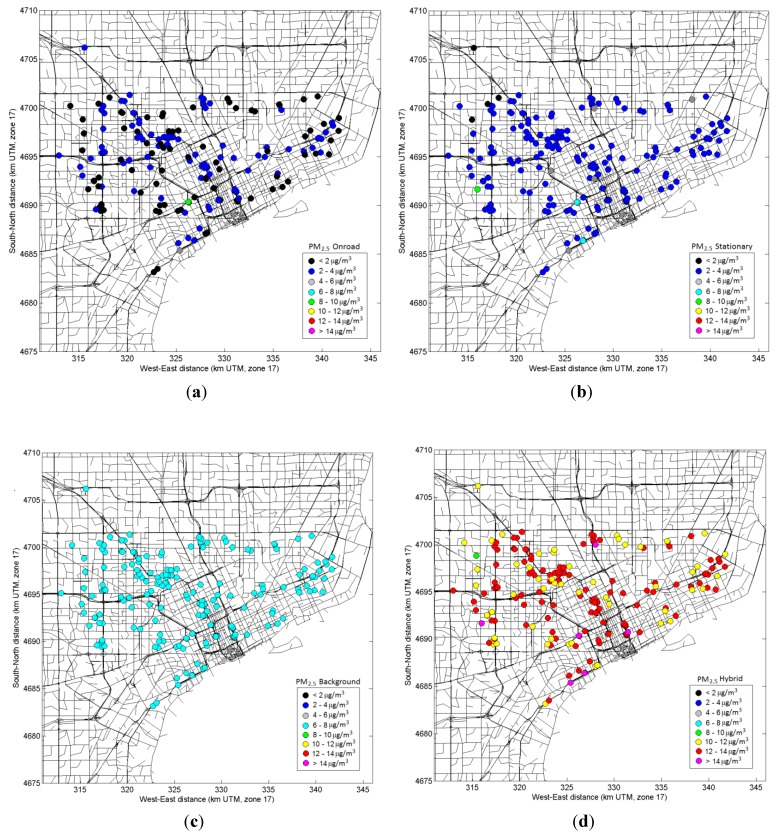
Spatial maps of modeled daily PM_2.5_ concentrations averaged during September–October 2010, showing contributions from mobile sources (**a**); stationary sources (**b**); urban background (**c**); and total (**d**).

The model-based exposure metrics for CO, NO*_x_*, PM_2.5_ and its components (EC and OC), were calculated from hourly predictions and were able to capture the spatial and temporal variability across the health study domain. The modeling approach also allowed estimating relative contributions of roadways *vs.* stationary sources and urban background. Figure 9 and Figure 10 show spatial maps of modeled daily NO*_x_* and PM_2.5_ concentrations averaged over the study period (September–October 2010) and the relative contributions of mobile sources, stationary sources, and urban background as well as the total (hybrid). For both NO*_x_* and PM_2.5_, the urban background was nearly uniform across the domain, while mobile source contributions varied across the domain–with higher concentrations next to major roadways and lower concentrations away from roads. The overall mobile source contribution, however, was not the same for NO*_x_* and PM_2.5_. For NO*_x_*, urban background contributes less than half of total concentrations, whereas for PM_2.5_, the urban background dominated and the local impact of mobile sources was less than 30%. Also, stationary source contributions for PM_2.5_ were of similar range to mobile sources.

## 4. Conclusions

Here we presented an application of a hybrid modeling approach to estimate exposure metrics in support of an urban scale epidemiologic study of exposures to traffic-related pollutants for children with asthma living near major roadways in Detroit, Michigan. The modeling approach involved the development and use of a detailed emissions inventory and multiple dispersion models to estimate ambient air pollution concentrations. The emissions inventory was based on a detailed geometry of the road network, traffic volumes, temporal allocation factors, fleet mix, and pollutant specific emission factors. These road-link emissions were used as inputs to RLINE, the newly developed dispersion model specifically designed for near-road applications. Thus, the model-based exposure metrics provided the temporal and spatial resolution needed for the epidemiologic study. Using a novel mini-grid approach, the modeling was able to resolve near-road air pollutant gradients. The hybrid modeling approach also provided an opportunity to compare relative contributions of various sources: stationary sources, roadways, urban background, and total. While near-road gradients of roadway emissions within 300 m were strong for all pollutants, their relative contributions to the total concentration varied by pollutant.

The hybrid modeling approach used in NEXUS provides new information regarding exposure to traffic-related air pollutants that is not captured by simpler exposure metrics (such as traffic intensity and distance to roads) commonly used in environmental epidemiology studies of traffic-related air pollution. Such additional information on strong spatial and temporal variation of pollutant concentrations and the relative contribution of various source categories to the total concentration could benefit future traffic-related health assessments. The hybrid modeling approach used in NEXUS could be also used for estimating exposures in other epidemiological studies where adequate measurements of traffic- or other source-related air pollutants are not feasible.

## References

[1] Health Effects Institute (HEI) (2010). Traffic-Related Air Pollution: A Critical Review of the Literature on Emissions, Exposure, and Health Effect.

[2] Cakmak S., Mahmud M., Grgicak-Mannion A., Dales R. (2012). The influence of neighborhood traffic density on the respiratory health of elementary schoolchildren. Environ. Int..

[3] Rosenbloom J., Wilker E., Mukamal K., Schwartz J., Mittleman M. (2012). Residential proximity to major roadway and 10-year all-cause mortality after myocardial infarction. Circulation.

[4] Chen H., Goldberg M., Burnett R., Jerrett M., Wheeler A., Villeneuve P. (2013). Long-Term exposure to traffic-related air pollution and cardiovascular mortality. Epidemiology.

[5] Gehring U., Gruzieva O., Agius R.M., Beelen R., Custovic A., Cyrys J., Eeftens M., Flexeder C., Fuertes E., Heinrich J. (2013). Air pollution exposure and lung function in children: The ESCAPE Project. Environ. Health Perspect..

[6] Miranda M., Edwards S., Chang H., Auten R. (2013). Proximity to roadways and pregnancy outcomes. J. Expos. Sci. Environ. Epidemiol..

[7] Batterman S., Burke J., Isakov V., Lewis T., Mukherjee B., Robins T. A comparison of exposure metrics for traffic-related air pollutants: application to epidemiology studies in detroit, michigan. Int. J. Environ. Res. Public Health.

[8] Wheeler A., Smith-Doiron M., Xu X., Gilbert N., Brook J. (2008). Intra-Urban variability of air pollution in Windsor, Ontario-measurement and modeling for human exposure assessment. Environ. Res..

[9] Matte T.D., Ross Z., Kheirbek I., Eisl H., Johnson S., Gorczynski J.E., Kass D., Markowitz S., Pezeshki G., Clougherty J.E. (2013). Monitoring intraurban spatial patterns of multiple combustion air pollutants in New York City: Design and implementation. J. Expos. Sci. Environ. Epidemiol..

[10] Karner A., Eisinger D., Niemeier D. (2010). Near-Roadway air quality: Synthesizing the findings from real-world data. Environ. Sci. Technol..

[11] Beckx C., Panis L.I., Uljee I., Arentze T., Janssens D., Wets G. (2009). Disaggregation of nation-wide dynamic population exposure estimates in The Netherlands: Applications of activity-based transport models. Atmos. Environ..

[12] Hatzopoulou M., Miller E.J. (2010). Linking an activity-based travel demand model with traffic emission and dispersion models: Transport’s contribution to air pollution in Toronto. Transp. Res. Part D Transp. Environ..

[13] McConnell R., Islam T., Shankardass K., Jerrett M., Lurmann F., Gilliland F., Gauderman J., Avol E., Kunzli N., Yao L. (2010). Childhood incident asthma and traffic-related air pollution at home and school. Environ. Health Persp..

[14] Gruzieva O., Bellander T., Eneroth K., Kull I., Melén E., Nordling E., van Hage M., Wicjman M., Moskalenko V., Hulchiy O. (2013). Traffic-Related air pollution and development of allergic sensitization in children during the first 8 years of life. J. Allergy Clin. Immunol..

[15] Sørensen M., Hoffmann B., Hvidberg M., Ketzel M., Jensen S.S., Andersen Z.J., Tjønneland A., Overvad K., Raaschou-Nielse O. (2012). Long-term exposure to traffic-related air pollution associated with blood pressure and self-reported hypertension in a Danish cohort. Environ. Health Persp..

[16] Sarnat S.E., Sarnat J.A., Mulholland J., Isakov V., Ozkaynak H., Chang H.H., Klein M., Tolbert P.E. (2013). Application of alternative spatiotemporal metrics of ambient air pollution exposure in a time-series epidemiological study in Atlanta. J. Expos. Sci. Environ. Epidemiol..

[17] Gurram S., Stuart A.L., Pinjari A.R. (2014). Impact of travel activity and urbanicity on exposures to ambient nitrogen oxides and on exposure disparities between sub-populations in Tampa, Florida. Air Qual. Atmos. Health.

[18] Byun D., Schere K. (2006). Review of the governing equations, computational algorithms, and other components of the models-3 Community Multiscale Air Quality (CMAQ) modeling system. Appl. Mech. Rev..

[19] Cimorelli A.J., Perry S.G., Venkatram A., Weil J.C., Paine R.J., Wilson R.B., Lee R.F., Peters W.D., Brode R.W. (2005). AERMOD: A dispersion model for industrial source applications. Part I: General model formulation and boundary layer characterization. J. Appl. Meteorol. Climatol..

[20] Cook R., Isakov V., Touma J.S., Benjey W., Thurman J., Kinnee E., Ensley D. (2008). Resolving local-scale emissions for modeling air quality near roadways. J. Air Waste Manag. Assoc..

[21] Dionisio K.L., Isakov V., Baxter L.K., Sarnat J.A., Sarnat S.E., Burke J., Rosenbaum A., Graham S.E., Cook R., Mulholland J. (2013). Development and evaluation of alternative approaches for exposure assessment of multiple air pollutants in Atlanta, Georgia. J. Expos Sci. Environ. Epidemiol..

[22] Isakov V., Touma J., Burke J., Lobdell D., Palma T., Rosenbaum A., Kozkaynak H. (2009). Combining regional-and local-scale air quality models with exposure models for use in environmental health studies. J. Air Waste Manag. Assoc..

[23] Vette A., Burke J., Norris G., Landis M., Batterman S., Breen M., Isakov V., Lewis T., Gilmour M.I., Kamal A. (2013). The Near-Road Exposures and Effects of Urban Air Pollutants Study (NEXUS): Study design and methods. Sci. Total Environ..

[24] Snyder M.G., Venkatram A., Heist D.K., Perry S.G., Petersen W.B., Isakov V. (2013). RLINE: A line source dispersion model for near-surface releases. Atmos. Environ..

[25] Venkatram A., Snyder M.G., Heist D.K., Perry S.G., Petersen W.B., Isakov V. (2013). Re-formulation of plume spread for near-surface dispersion. Atmos. Environ..

[26] U.S. Environmental Protection Agency The 2008 National Emissions Inventory. http://www.epa.gov/ttn/chief/net/2008inventory.html.

[27] Arunachalam S., Valencia A., Akita Y., Serre M., Omary M., Garcia V., Isakov V. Estimating regional background air quality using space/time ordinary kriging to support exposure studies. Int. J. Environ. Res. Public Health.

[28] Snyder M.G., Arunachalam S., Isakov V., Talgo K., Naess B., Valencia A., Davis N., Cook R. Creating mobile source emissions for an urban-scale air quality assessment to support exposure studies. Int. J. Environ. Res. Public Health.

[29] Houyoux M.R., Vukovich J.M., Coats C.J., Wheeler N.J.M. (2000). Emission inventory development and processing for the Seasonal Model for Regional Air Quality (SMRAQ) project. J. Geophys. Res..

[30] Lobdell D.T., Isakov V., Baxter L., Touma J.S., Smuts M.B., Özkaynak H. (2011). Feasibility of assessing public health impacts of air pollution reduction programs on a local scale: New Haven case study. Environ. Health Persp..

